# The nature of thallium crystals in *Brassica oleracea* (kale): a synchrotron multi-technique investigation

**DOI:** 10.1093/mtomcs/mfag010

**Published:** 2026-02-23

**Authors:** Amelia Corzo-Remigio, Hugh H Harris, Michael W M Jones, Tony Wang, Dennis Brueckner, Kathryn M Spiers, Jan Garrevoet, Antony van der Ent

**Affiliations:** Centre for Environmental Responsibility in Mining, Sustainable Minerals Institute, The University of Queensland, Brisbane, Australia; Discipline of Chemistry, The University of Adelaide, Adelaide, Australia; Central Analytical Research Facility, Queensland University of Technology, Brisbane, Australia; School of Chemistry and Physics, Queensland University of Technology, Australia; Central Analytical Research Facility, Queensland University of Technology, Brisbane, Australia; Deutsches Elektronen-Synchrotron DESY, Hamburg, Germany; Deutsches Elektronen-Synchrotron DESY, Hamburg, Germany; Deutsches Elektronen-Synchrotron DESY, Hamburg, Germany; Laboratory of Genetics, Wageningen University and Research, Wageningen, The Netherlands

## Abstract

*Brassica* cultivars have the ability to hyperaccumulate thallium when growing on soils contaminated with this element. Earlier research identified the presence of crystalline thallium deposits in the leaves of *Brassica oleracea* var. *acephala*. The aim of this study was to investigate the nature of these thallium crystals. A combination of synchrotron-based methods was used involving micro-X-ray fluorescence (µXRF) for the elemental distribution in the leaves and micro-X-ray diffraction mapping (µXDM) for the identification of the crystals. Thallium concentrates along the foliar margins, especially near vascular bundles, and dense congregations of minute thallium-rich crystals are observed in these areas. The thallium speciation is revealed to be nearly exclusively monovalent thallium, whilst the crystals are largely cubic thallium chloride, TlCl_(s)_. The formation of thallium-rich crystals in the form of thallium chloride appears to be a tolerance mechanism similar to that of halophytes, and possibly a way in which excess thallium is expelled from leaves.

## Graphical Abstract

**Graphical Abstract:**
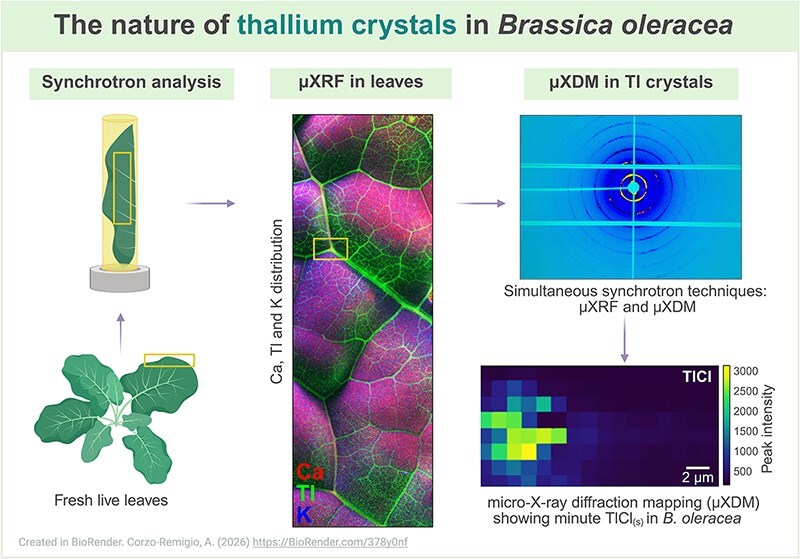

## Introduction

The hyperaccumulation phenomenon occurs in several species from the Brassicaceae family with some of the strongest hyperaccumulators for zinc (Zn) and thallium (Tl) belonging to this family: *Noccaea caerulescens* [1], and *Biscutella laevigata* [2], respectively. While zinc is a micronutrient essential for plants, thallium is non-essential and extremely toxic for living organisms because of the chemical resemblance to the essential macronutrient potassium (K^+^) [3, 4]. The threshold for Tl hyperaccumulation in plants has been set at 100 µg g^−1^ in above ground tissues [5]. Until now, only six species are known to be Tl hyperaccumulators with two of them belonging to the Brassicaceae family, *Biscutella laevigata* (32 700 µg Tl g^−1^ in their leaves) [2] and *Iberis intermedia* (4000 µg Tl g^−1^) [6]. Even though Tl is a toxic metal categorized as an emergent pollutant [7], it is used in isotopes in medical applications, optical glasses, and in superconductors, making it an economically valuable metal at prices of 8800 US$ per kilogram [8]. Therefore, the use of hyperaccumulator plants to remove Tl from contaminated soil has a potential economic benefit through sustainable methods, such as phytoextraction [9]. For instance, based on an estimated 4 t of *Biscutella laevigata* biomass with 425 µg Tl g^−1^ [10], and 50 plants per m^2^, Tl yields of 1.7 kg ha^−1^ yr^−1^ would generate 14 960 US$; a similar calculation for *Iberis linifolia*, based on an estimated 10 t of biomass with 800 µg Tl g^−1^ [11], would produce Tl yields of 8 kg ha^−1^ yr^−1^ valued at US$70 400 in both cases excluding production and processing/refining costs (which will no doubt be substantial).

*Brassica* cultivars can bioaccumulate Tl from soils contaminated with Tl through natural processes, or as a result of mining or industrial activities [12]. For example, kale (*Brassica oleracea* var. *acephala*) grown in soils containing 0.1–124 µg Tl g^−1^ had higher bioconcentration factors (0.06) in edible parts compared to other vegetables, and as consequence ranked as the highest health risk to humans [13]. Even though the Tl hypertolerance and hyperaccumulation of *Brassica* crops is of great concern for human health, this trait is also a possibility for the application of phytoremediation. Indian mustard (*Brassica juncea*), the most geographically distributed Brassicaceae plant, can tolerate high Tl concentrations with the potential benefits of phytoextraction and bioprospecting [14]. Furthermore, the availability of *Brassica* seeds, and the rapid growth of plants in the genus enables the study of the mechanisms and pathways involved in the transport and storage of Tl.

Research on the mechanisms and pathways involved in the transport and storage of Tl in plants has significantly increased [15, 16]. Thallium exists in nature in two oxidation states, Tl(I) is more stable and abundant compared to Tl(III) [17], albeit that the latter is 50 000 times more toxic to living organisms like phytoplankton (unicellular algae, *Chlorella* sp.) [18]. In the hyperaccumulators *Biscutella laevigata* and *Iberis intermedia*, synchrotron studies revealed the predominance of Tl(I) in plant tissues. In *Brassica oleracea* var. *acephala* Tl(I) was found as the predominant species [19]. A study on isotopic fractionation validates these results [14]. Thallium is stored on the margin of the leaves, and it is hypothesized that plants use evapotranspiration channels to expel excess Tl *via* hydathodes and minute Tl-rich crystals were observed [19]. This mechanism is also used by their affinities the halophytes, forming salt crystals through salt glands on the leaves; for example, in *Armeria maritima* ssp. *halleri*, crystals rich in copper and other elements were reported [20]. In addition, in a lead (Pb) tolerant population of this same species, crystals with up to 31.74% wt Pb were reported [21]. Metal tolerant plants store excess metals by forming oxalate crystals in the leaves, which was observed in *Gomphrena claussenii*, where cadmium is bound predominantly to oxygen ligands (with 88% Cd—O—C coordination) with the remainder bound by sulfur donor ligands (Cd—S—C coordination) [22]. Lead crystals were mostly observed in the roots rather than in the stem or leaves of *Zea mays* L. In the roots, Pb initially precipitates in the organelle dictyosome and is then mobilized to the surface and extruded from the cell walls [23]. Plants often compartmentalize toxic elements in tissue areas that will not affect their physiological functions, and this may be the case for Tl sequestration in *Brassica oleracea*. However, unresolved questions remain. What is the composition of these Tl-crystals observed in an earlier study? How does the formation of these Tl-crystals contribute to Tl tolerance? What are the implications of these Tl-crystals for food safety or for phytoextraction?

Synchrotron-based X-ray microscopy (XFM) enables cutting-edge microanalytical technique allowing quantitative mapping of elemental distribution with high sensitivity [24, 25]. The experimental setup of XFM enables a range of different techniques to be used in the same experiment, including micro-X-ray fluorescence (μXRF) for elemental imaging and micro-X-ray diffraction mapping (μXDM) for elucidation of crystal phases [26]. Scanning X-ray diffraction enables to obtain X-ray diffraction traces in spatial mapping of crystallinity within a sample [27]. Due to the highly localised nature and small size of crystalline sequestration of toxic metals in plants [19–23, 28], the brilliant source and focused beam available in the combination of synchrotron-based XFM techniques make it uniquely suitable for the analysis of such crystalline deposits in plants.

The combination of the aforementioned suite of synchrotron XFM techniques can yield powerful insights into the identity of crystalline deposits [26, 27] but to our knowledge, has never been attempted simultaneously for plant samples. The aim of this study was to investigate the nature of Tl crystals in *Brassica oleracea* var. *acephala* fresh hydrated leaves using µXRF to map the elemental distribution in the leaves and µXDM to identify the crystal phases of Tl. This information can be used to better understand the ecotoxicology of these edible crops, and to either limit the uptake of toxins or conversely to exploit the potential of these cultivars for phytoextraction of Tl polluted soils.

## Materials and methods

### Plant culture conditions for thallium exposure in hydroponics

Seeds of *Brassica oleracea* var. *acephala* were surface sterilised in 4% sodium hypochlorite solution (10 min.) then rinsed and germinated on sterile MS medium gel in closed containers at 25°C. After the cotyledons had fully emerged, seedlings were then further cultivated in a growth chamber with 12/12-hr light/dark cycle. Growth light was provided using high-intensity LEDs with photosynthetically active radiation (PAR) (VYPRx PLUS with PhysioSpec Indoor spectrum, Fluence Bioengineering, Austin, TX, USA), with a photosynthetic photon flux density of 700 μmol m^2^ s^−1^ and a day/night temperature regime of 25/20°C. Five seedlings were then transferred to hydroponic cultures. The hydroponics experiment was conducted in four containers (11 × 30 × 40 cm; capacity ∼12 L). In each treatment, 5 plants were grown in 3 cm round plastic baskets with a foam disc so that the roots could be immersed in the nutrient solution. The modified ½ strength Hoagland’s solution had the following composition : K (3 mM as KNO_3_), Ca (2 mM as Ca(NO_3_)_2_·4H_2_O), P (1 mM as NH_4_H_2_PO_4_), N (8 mM as KNO_3_, Ca(NO_3_)_2_·4H_2_O, and NH_4_H_2_PO_4_), Mg (0.5 mM as MgSO_4_·7H_2_O), Fe (40 µM as Fe(K)-HBED), Cl (1 µM as KCl), B (25 µM as H_3_BO_3_), Mn (2 µM as MnSO_4_·4H_2_O), Zn (2 µM as ZnSO_4_·7H_2_O), Cu (0.1 µM CuSO_4_·5H_2_O), Mo (0.1 µM as Na_2_MoO_4_·2H_2_O) and 2 mM MES (2-(N-morpholino)ethanesulfonic acid) buffer adjusted to pH 5.8 with KOH. Plants were first cultivated without treatment for 24 days and then exposed to 7.5 µM Tl as Tl(NO_3_)_2_, a dose in which *Brassica oleracea* can grow without experiencing major toxicity symptoms or growth reduction [19]. The solutions were aerated with air-stone diffusers at the bottom of each container. The nutrient solutions were changed completely once a week. The plants were harvested after 30 days experimental period, rinsed with de-ionised water, and divided into leaves and roots using scissors.

### Bulk elemental analysis of plant samples

The plant material was dried in a dehydrating oven at 60°C for at least 48 hours, then ground to a fine powder (<200 µm) in an impact mill (IKA TubeMill 100 Control) and subsamples (0.5 g) were inserted into custom XRF sample holders and covered with a 6 µm polypropylene thin film (Chemplex Industries Inc.) for XRF analysis. The bulk XRF analysis of the powdered plant material was performed using a Z-Spec JP500 instrument (Z-Spec Inc.). This instrumentation uses monochromatic X-ray fluorescence excitation at 17.48 keV to analyse elements Z = 13 (Al) to *Z* = 39 (Y) on the K-lines and up to *Z* = 92 (U) on the L-lines with optimum sensitivity for elements Cu-Se and Hg-Tl-Pb with LODs ranging from 0.009–0.025 mg kg^−1^ [29, 30]. Samples were analysed for 30 sec. in the Plant Mode. Quality controls included NIST SRM 1570a Trace elements in spinach leaves and NIST SRM 1573a Tomato leaves (See Supp Table S1 for recoveries).

### Synchrotron XFM experiments

**Figure 1:**
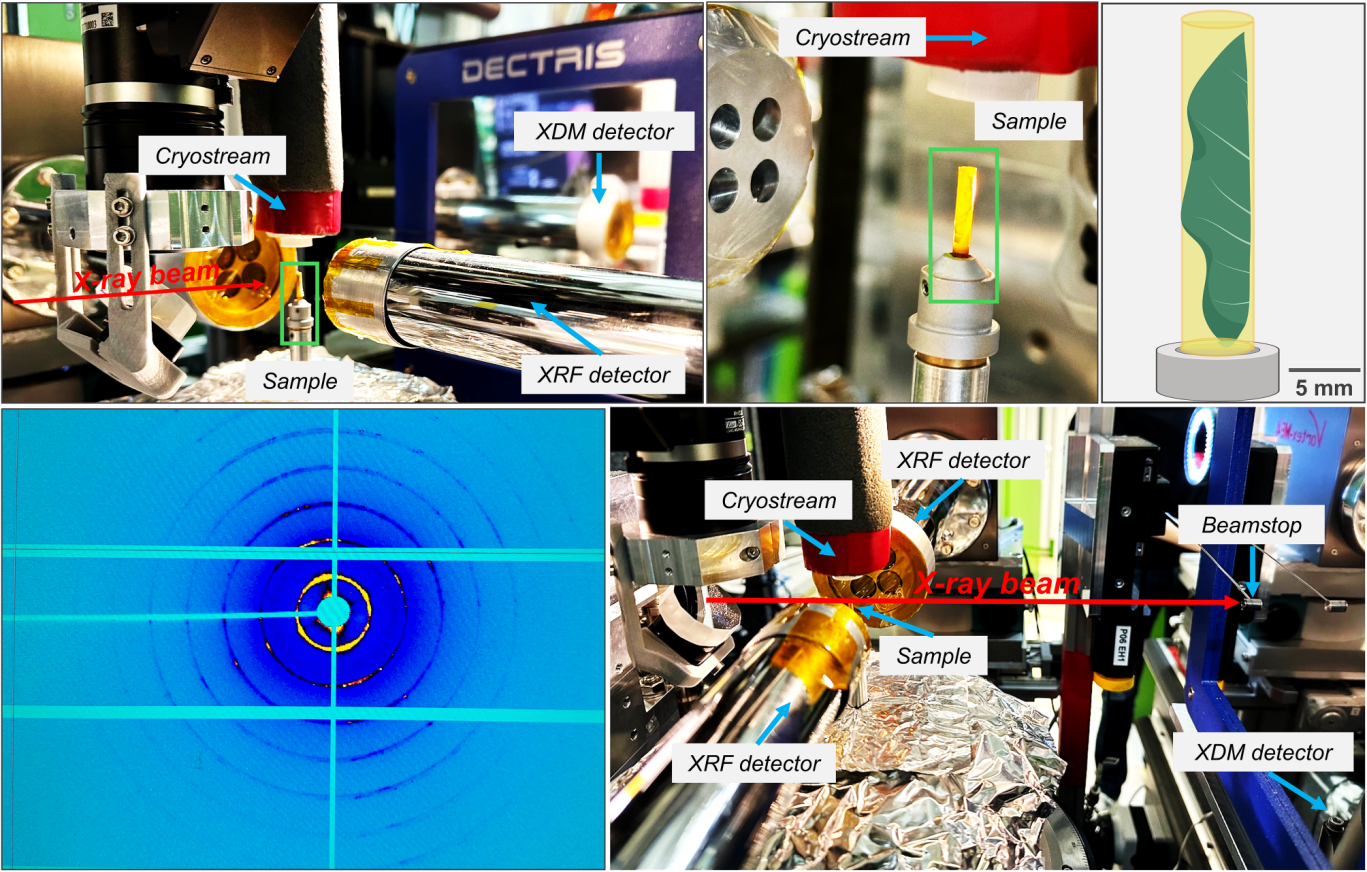
Experimental conditions of the synchrotron experiment involving simultaneously micro-X-ray fluorescence (µXRF) and micro-X-ray diffraction mapping (µXDM).

The XFM experiments were conducted at PETRA III, part of DESY (Deutsches Elektronen-Synchrotron), a 6 GeV source of synchrotron radiation, whereby the hard X-ray microprobe undulator beamline P06 was used [31, 32]. More information on instrumental parameters and setup have been presented in previous research [33]. The XRF mapping and transmission XRD experiments (see Fig. 1) were conducted at an X-ray photon energy of 13 keV. KB mirrors were used to focus the beam down to 680 × 510 nm (h × v) with a flux of about 1.6^10^ ph/s in focus. XRF signal was recorded using two four-element Vortex SDDs, placed at 90° and 270° relative to the X-ray beam direction [34]. The diffraction data were collected using a Pilatus 1 M pixel detector (981 × 1043 pixels, pixel size: 172 × 172 μm^2^) with the direct beam aiming in the centre of the detector, where a beam stop was placed. The PONI positions (including *approx*. 0.21 m Sample to Detector Distance and other detector tilts etc.) of the 2D pixel detector were calibrated according to a summed 2D diffraction frame of a LaB_6_ standard using *pyFAI-calib2* [35, 36]. The 2D frames of the entire scans across the sample regions of interests were radially batch integrated into 1D diffraction data according to this calibration using *pyFAI-integrate* [35, 36]. The samples were frozen *in situ* within 10 minutes after excision, at the beamline sample position under a cryostream operated at -140°C (Oxford Cryosystems 700 Series Cryostream Cooler, Oxford, UK) and remained under the cryostream for the full duration of the measurement to cool the sample and eliminate potential radiation damage [37].

### Data processing

The data acquisition was handled by a custom workflow [32] and the XRF data were processed using non-linear least squares fitting as implemented in PyMCA [38]. A Se-foil with known areal density was used as reference for quantification. This resulted in 32-bit .tiff files with pixel values corresponding to the areal density of each element in µg cm^−2^. ImageJ was used to create the figures, changing LUT to ‘Fire’, adjusting the maximum values, using the ‘Calibration’ tool to add the modelled concentration bars and adding length scales [39].

## Results

### Revealing plant and tissue-level distribution of thallium (µXRF imaging)

**Figure 2:**
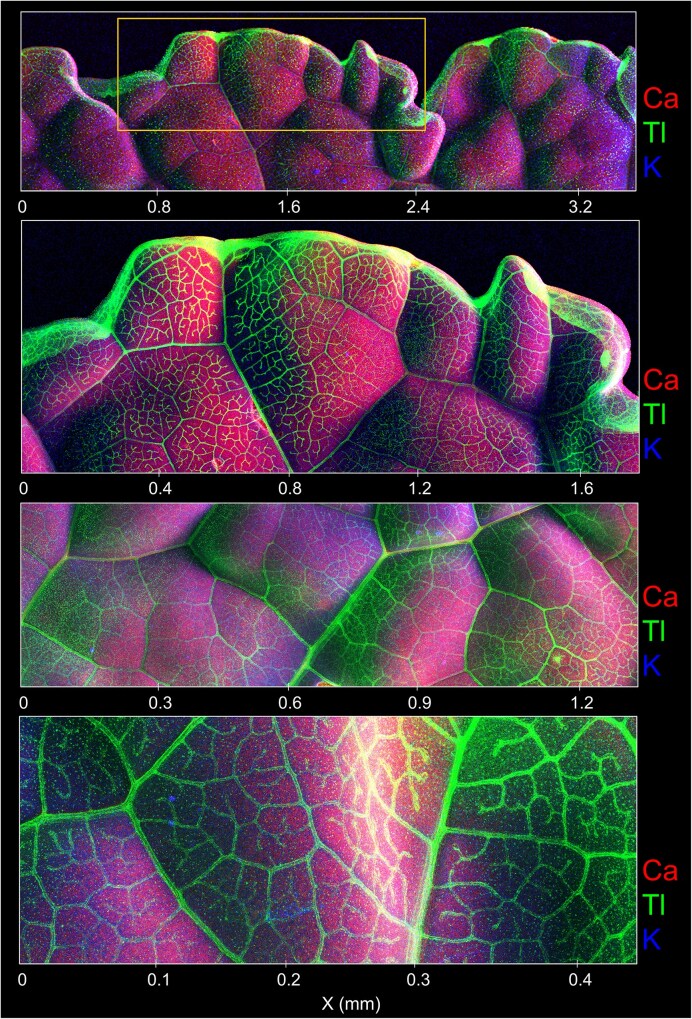
Synchrotron-based μXRF elemental maps of Ca, K and Tl in different areas of the same leaf of *Brassica oleracea* var. *acephala*, kale, Red Russian cultivar. Overview scan and several detailed areas. The total acquisition time for the scans was 7 minutes each, with a dwell time of 5 ms, and 10 µm resolution. The scale of each detail map is shown at the bottom in mm.

**Figure 3:**
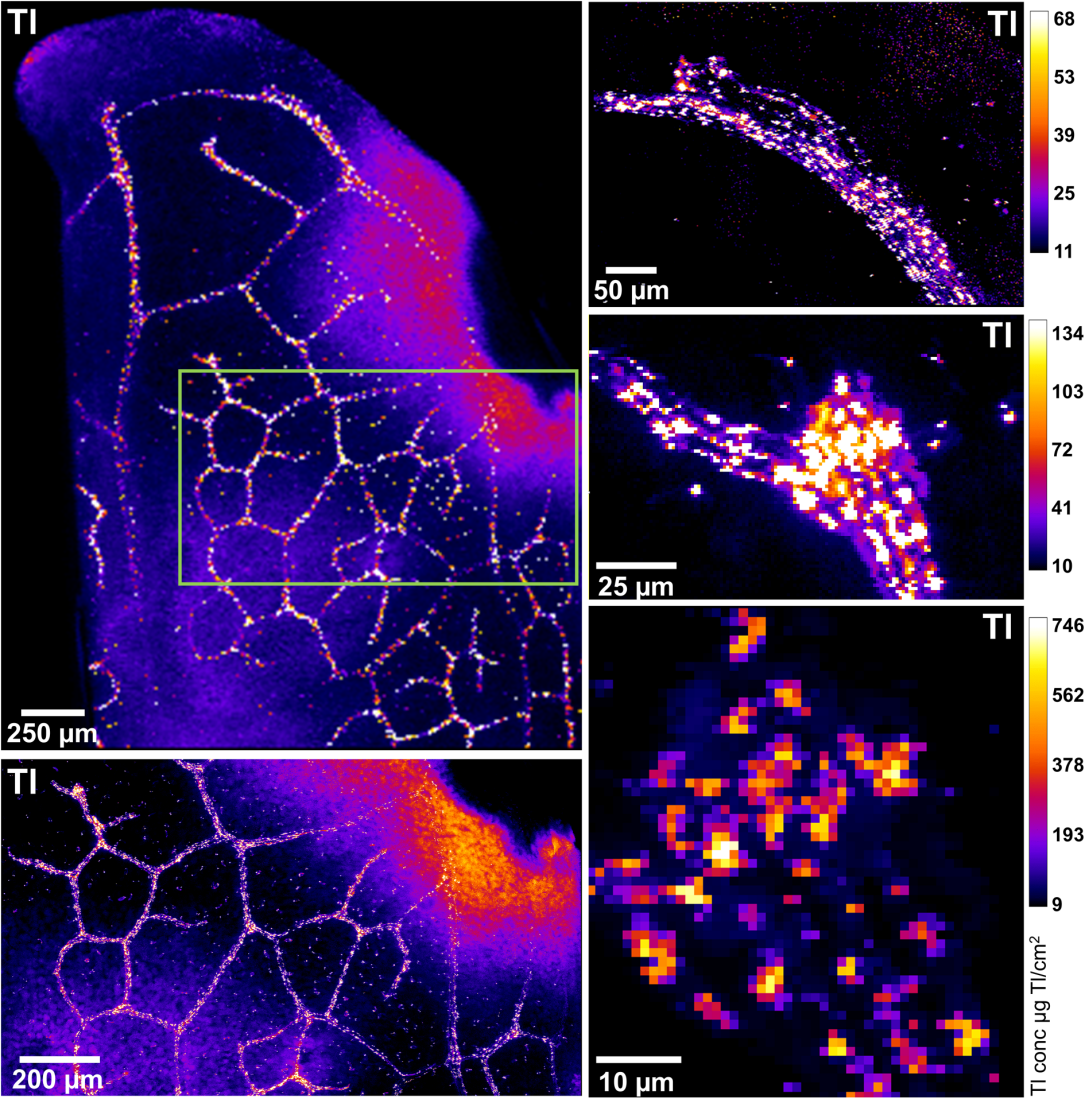
Synchrotron-based μXRF elemental maps of Tl of *Brassica oleracea* var. *acephala*, kale, Red Russian cultivar showing from low to higher magnification (green rectangle) how Tl is distributed near the veins of the leaves forming crystalline structures. The total acquisition time for the scans were between 1 and 155 minutes, with a dwell time of 5 ms (for most of the maps), and 1000 ms (for the last image bottom-right). The resolution for the top-left map was 10 µm and 1 µm for the rest of the maps.

The elemental distribution in *Brassica oleracea* var. *acephala*, Red Russian cultivar, is presented in Figs. 2 and 3, and Supplementary Figs (S1-S3), and the bulk elemental analysis in Table 1. For comparison reasons, other elements were also considered in the elemental distribution gathered with the µXRF. Thallium was mainly distributed in the margins of the leaves, and specially on the vascular bundles, and this differs greatly from the distribution of calcium and potassium, which were evenly distributed on the leaves (Fig. 2, and Fig. S1-S3). Thallium distribution was similar to that of microelements such as manganese, iron and zinc (Fig. S1-S3). A magnified image of Tl distribution around the veins is presented in Fig. 3, where formations of microcrystals can be observed in the veins. Highly Tl enriched minute regions (5 µm) were also identified in *Brassica oleracea* var *acephala*, Nero di Toscana cultivar (Fig. S4). Thallium concentrations in the shoots of *Brassica oleracea* var *acephala* reached up to 6280 µg g^−1^, which is considerably higher to root concentrations ranging from 1720—1840 µg g^−1^ (Table 1).

**Table 1:** Element concentration (µg g^−1^) of shoot and roots of *Brassica oleracea* var *acephala* (kale) analysed with MXRF (JP500 instrument, Z-Spec Inc).

Plant section	Ca	Mn	Fe	Cu	Zn	Tl
Kale shoot 1	47 000	193	92.9	6.53	77.6	6000
Kale shoot 2	52 300	253	101	7.42	69.9	6280
Kale shoot 3	54 500	210	97.5	7.02	71.6	6220
Kale root 1	28 600	500	1440	175	260	1720
Kale root 2	23 600	501	1740	188	259	1750
Kale root 3	24 300	508	2020	192	261	1840

### Identifying crystalline phases of thallium-rich crystals (μXDM analysis)

**Figure 4:**
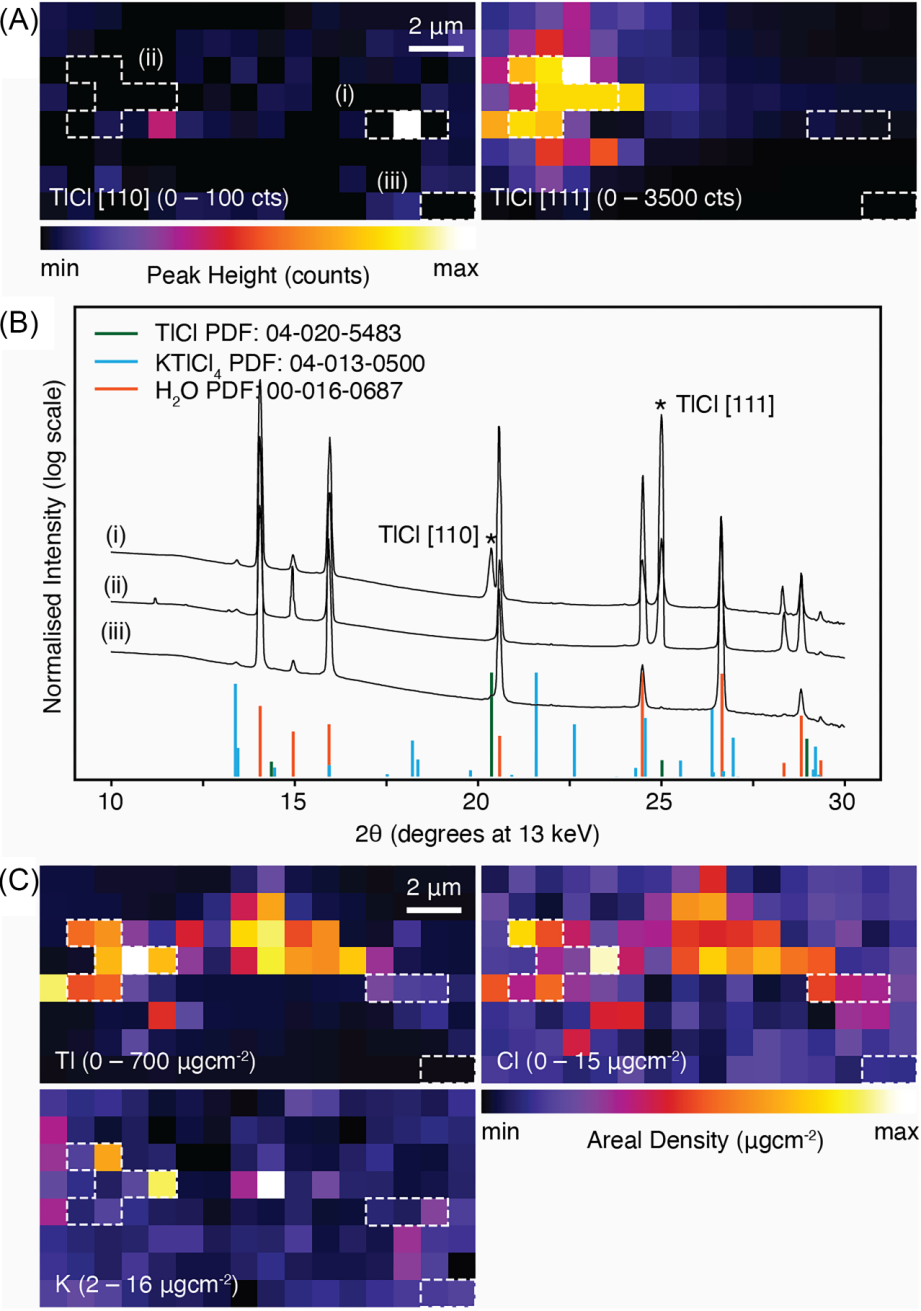
Simultaneous synchrotron-based mapping of *Brassica oleracea* var. *acephala*, kale, Red Russian cultivar: (A) micro-X-ray diffraction mapping (µXDM) showing identified peaks of thallium chloride (TlCl); (B) micro-XRD spectra from selected regions in (A) identified with white dashed boxes show the presence of TlCl and KTlCl_4_ peaks; (C) μXRF elemental maps of Tl, Cl, and K. Additional elemental maps are presented in Supp Fig. S8. The total acquisition time for the scan was 3 minutes, with a dwell time of 1000 ms, and 1 µm resolution. Scale bars denote 20 µm.

**Figure 5:**
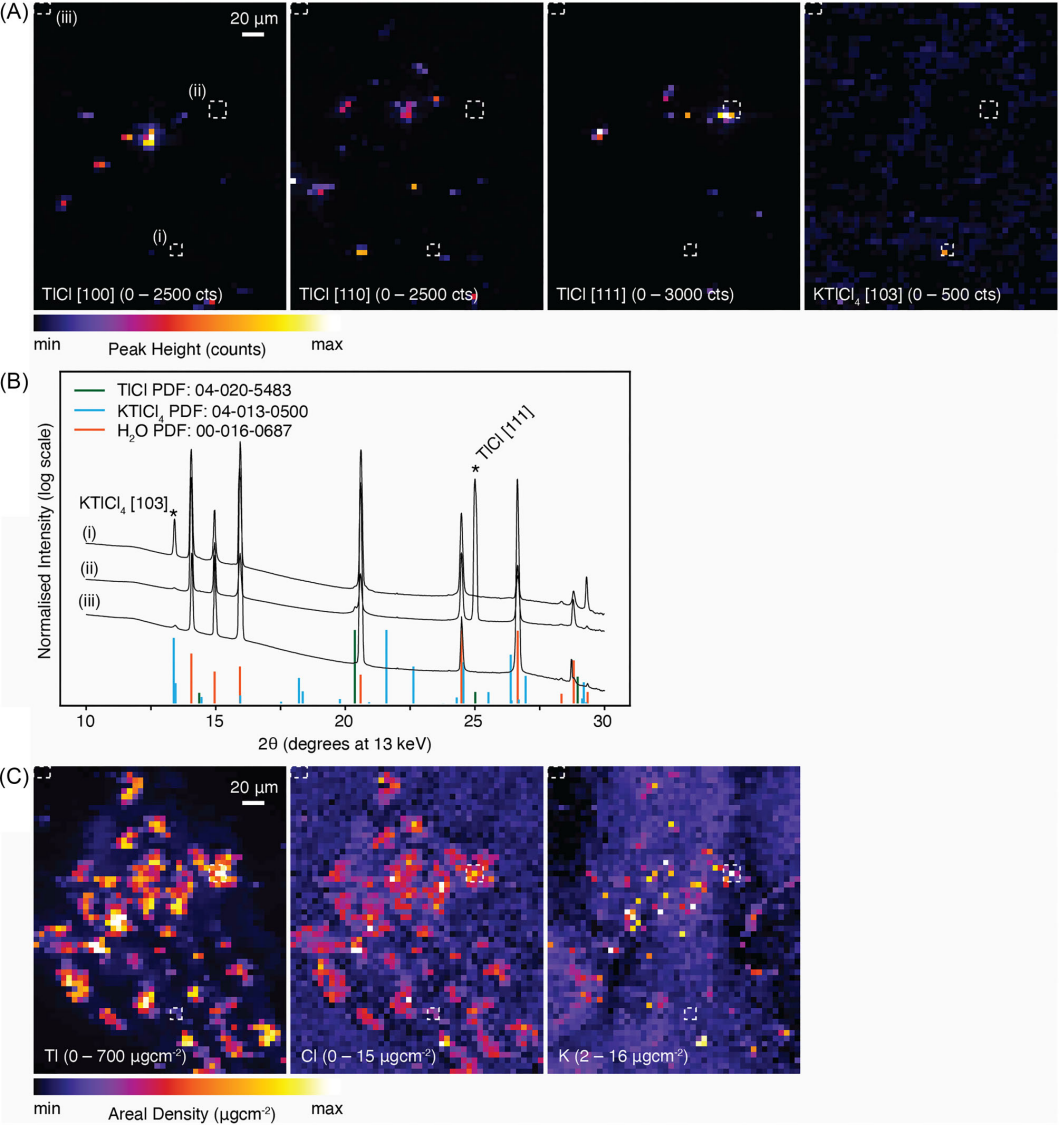
Simultaneous synchrotron-based mapping of *Brassica oleracea* var. *acephala*, kale, Red Russian cultivar: (A) micro-X-ray diffraction mapping (µXDM) showing identified peaks of thallium chloride (TlCl); (B) μXRD spectra from selected regions in (A) identified with white dashed boxes show the presence of Tl Cl peaks; (C) µXRF elemental maps of Tl, Cl, and K. Additional elemental maps are presented in Supp Fig. S9. The total acquisition time for the scan was 55 minutes, with a dwell time of 1000 ms, and 1 µm resolution. Scale bars denote 2 µm.

Supplementary Figs S5-S7 show the phase identification results of a typical summed integrated 1D diffraction data conducted in DIFFRAC.EVA v7 with respect to the ICDD PDF-5 + database. Other than the inevitable ice phase (PDF# 00-016-0687) due to the cryostream, a cubic phase thallium chloride (TlCl_(s)_, PDF# 04-020-5483) was identified, with diffraction peaks summarised in Table 2. A low angle, low intensity peak at 13.4° 2θ might suggest a KTlCl_4_ phase (PDF# 04-13-0500). A second low intensity peak at 20.9° 2θ identified only in the analysis of individual patterns (Fig. 4B, 5B, 6B) suggests the presence of a KTlCl_4_ phase. The Rietveld full pattern refinement (Supp Fig. S6) conducted using DIFFRAC.TOPAS v7 verified the presence of the cubic TlCl_(s)_ phase with a refined lattice parameter of TlCl_(s)_*a* = 3.8143(9) Å in agreement with other low temperature TlCl_(s)_ PDF cards (PDF# 04-002-0353, PDF# 04-018-3893). Following the full pattern refinement, calibrated diffraction data corresponding to each sample X-Y position was similarly radially averaged separately. The background was removed with 10 iterations of the SNIP algorithm [40] and a set of Gaussian peaks fitted simultaneously to the 2θ angles corresponding the identified TlCl_(s)_, and the potential KTlCl_4_ peaks (Table 2) together with the ice peaks with the 2θ range from 12° to 26° (Supp Fig. S7). Individual peaks where the fitted peak was over 2-fold the width identified in the ice peaks, or where the fitted intensity was less than a single count, were deemed to be fitting noise and were discarded. The intensity of each fitted peak was then spatially mapped to the sample X-Y position. Maps that contained no fitted TlCl or KTlCl_4_ peaks were discarded and are not shown. Technically, a single XRD peak does not allow an identification of crystalline phase. However, in micro-diffraction, it is not un-common that only a certain hkl peak was observed, especially when the crystal is bigger than the beam.

**Table 2:** Matched XRD peaks of the identified phases in *Brassica oleracea* var.*acephala*, kale, Red Russian cultivar.

Phases	Miller index	Matched 2θ Angle (°)
KTlCl_4_^a^	1 0 3	13.410
KTlCl_4_^a^	2 1 3	20.916
TlCl^b^	1 0 0	14.376
TlCl^b^	1 1 0	20.367
TlCl^b^	1 1 1	24.987

a PDF # 04-013-0500, possibly

b PDF# 04-020-5483

### Simultaneous μXDM and µXRF images of thallium rich crystals

**Figure 6:**
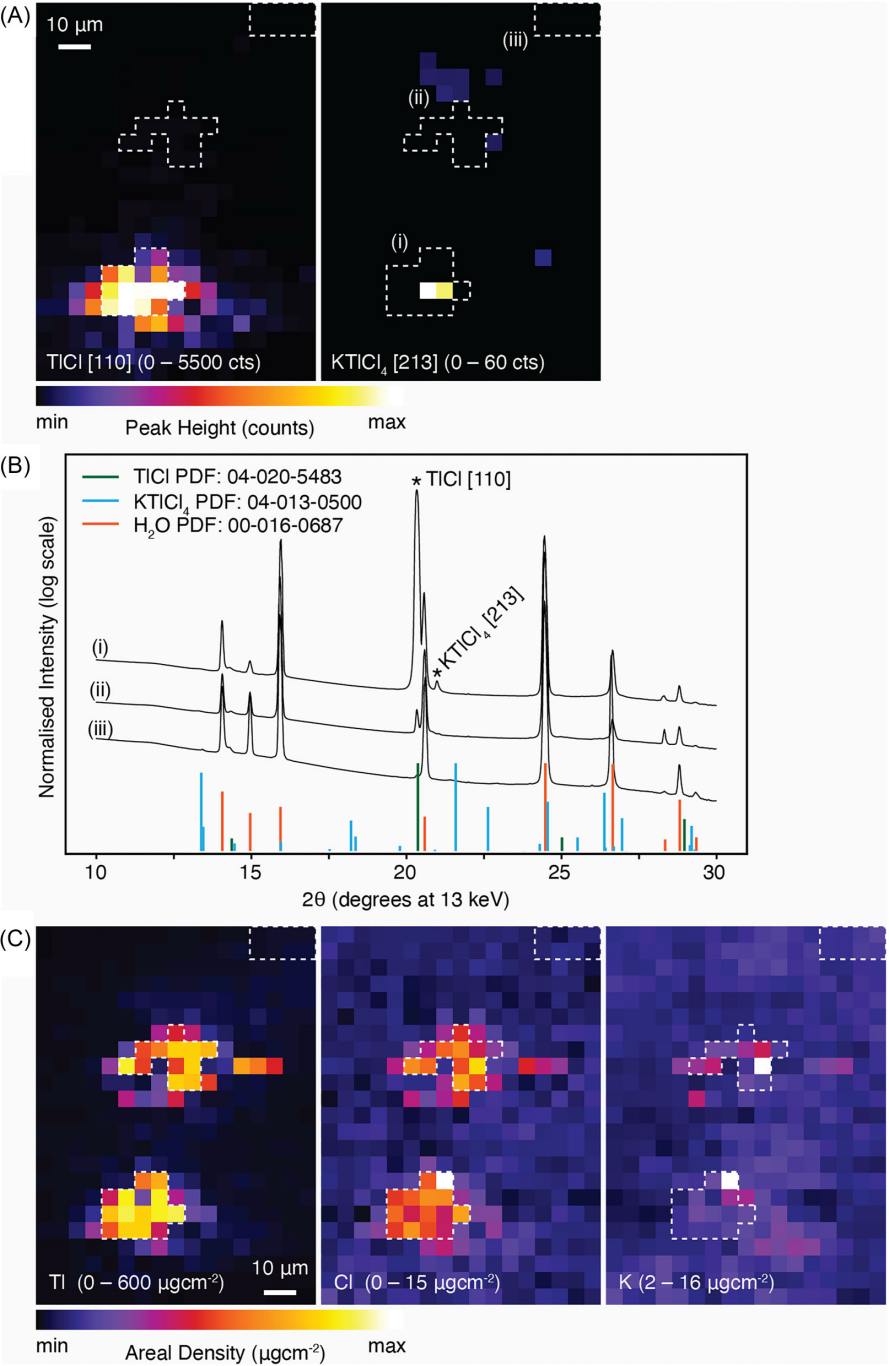
Simultaneous synchrotron-based mapping of *Brassica oleracea* var. *acephala*, kale, Red Russian cultivar: (A) micro-X-ray diffraction mapping (µXDM) showing identified peaks of thallium chloride (TlCl); (B) micro-XRD spectra from selected regions in (A) identified with white dashed boxes show the presence of TlCl and KTlCl_4_ peaks; (C) μXRF elemental maps of Tl, Cl, and K. Additional elemental maps are presented in Supp Fig. S10. The total acquisition time for the scan was 7 minutes, with a dwell time of 1000 ms, and 0.5 µm resolution. Scale bars denote 10 µm.

Images collected from simultaneous μXDM and µXRF mapping of *Brassica oleracea* var. *acephala*, Red Russian cultivar, are presented in Figs. 4–6. The cubic phases identified with the μXDM were compared against the elemental distribution gathered with the µXRF. The highest Tl concentration in the µXRF maps coincide with the enrichments in Cl and with the location of TlCl(_s_) identified through µXDM (Figs 4, 5, 6). Furthermore, each cluster of TlCl identified using µXDM corresponds to a single reflection indicating that each cluster represents a single TlCl crystal (for example the TlCl [111] peak in Fig. 4A and the TlCl [110] peak in Fig. 5A and 6A). In each of the cases the TlCl crystals are ∼5 µm in size.

## Discussion

This study revealed the Tl hyperaccumulation and the formation of TlCl_(s)_ crystals (∼5 µm) in *Brassica oleracea* var. *acephala*, Red Russian cultivar. The higher Tl enrichment in minute Tl-rich crystals has been previously observed in freeze-dried samples of the same plant species, and in fresh live samples of *Biscutella laevigata* [28], with X-ray absorption near edge structure (XANES) analysis revealing Tl(I) [19]. The formation of TlCl_(s)_ in fresh live *B. oleracea* var *acephala* can potentially be explained with the similarity of K(I) and Tl(I) [41]. We hypothesize that Tl enters to the plant using K^+^ channels, then is transported from roots to the shoots, where it is compartmentalized, predominantly in vacuoles, along with Cl, to maintain safe levels in the cytoplasm. Even though Tl hyperaccumulators tolerate high Tl levels, for homeostasis purposes the plant triggers mechanisms to expel the Tl excess. This process is not unlike that of many halophytes expelling excess salts (Na^+^, Cl^−^, and K^+^) through glands and epidermal bladder cells (EBC) [42, 43]. The salt bladder cells originate from the epidermis and enclose a huge central vacuole, where salts are stored, and eventually released [44], when excess of K^+^, Cl^−^, and Na^+^ becomes toxic [45]. The EBC also store metabolic compounds such as malate, flavonoids, cysteine, pinitol, inositol and calcium oxalate crystals [46] to balance the osmotic potential of the accumulated salts in the vacuoles [47]. Halophytes and hyperaccumulator plants might share the same mechanisms to cope with metabolic stress, and some halophytes can even accumulate metals [48]. The same plant structures the in leaves can excrete salts and metals, as shown in *Tamarix Smyrnensis* Bunge [49, 50]. Our findings evidence the similar mechanisms of halophytes and hyperaccumulator plants to cope with the excess of particular elements; nevertheless, there are still a myriad of questions underlying the specific mechanisms of Tl transport, compartmentation and regulation in plants. It is likely that molecular genetic techniques are required to provide answers to these questions.

Our study also identified Tl(III) in the form of KTlCl_4_ although only as a weak peak. Other supporting evidence, *e.g*. Tl chemical valence confirmation using Tl XANES data is required; previous research using XANES showed a minor proportion (less than 5%) of Tl(III) also in kale [19]. A speciation study using in *Sinapsis alba* L. reported Tl(III) of 10% of total Tl content using high-performance liquid chromatography (HPLC) and inductively coupled plasma mass spectrometry (ICP-MS) [51]. A latter study validated the prior results showing that Tl(I) oxidation to Tl(III) was not observed, on the contrary Tl(III) extract was reduced to Tl(I) in the presence of UV radiation [52]. Even though Tl(III) is more toxic than Tl(I), the trivalent oxidation state forms more stable complexes with organic ligands [52] and this is not uncommon in plants as the most toxic form of arsenic (As), As(III) is also stored in hyperaccumulator ferns to cope with As toxicity, as As(V) similarity with phosphate can lead to interference in ATP-related processes [53].

Thallium enrichment in *B. oleracea* var *acephala*, Red Russian cultivar, was found mainly in the veins, and for the Nero di Toscana cultivar in the borders corroborating previous research in *Brassica* crops, where higher Tl concentrations were reported in the margins and the apex of the same species, probably induced by guttation through hydathodes, and transpiration through the stomata, where crystals with 1.7 wt % Tl, 3 µm in size, where observed in guard cells of freeze dried samples [19]. This mechanism is not shared by Tl tolerant plants; for instance, the strongest Tl hyperaccumulator *Biscutella laevigata*, Les Malines accession, concentrate Tl evenly distributed in old leaves, and cross sections showed that Tl is mostly localized in the vacuoles of epidermal and mesophyll cells, and in basal trichome cells, with minute specks with the appearance of solid crystalline deposits with approximately 40 wt% Tl, 3–5 µm in size [28]. While in the Tl hyperaccumulator *Silene latifolia*, Tl is mainly enriched in the base of the midrib and depleted towards the apex [54].

In this study we report elevated Tl concentrations in edible parts of *B. oleracea* var *acephala*, kale, with up to 6280 µg g^−1^. Even though Tl is more toxic than the well-known poison arsenic, there is still a paucity on the development of guidelines regarding the safe levels in food, and only a few countries have advanced on this issue. Health Canada stated that food consumption is the primary source of Tl exposure [55]. The soil quality guideline for Tl in Canada is 1 mg kg^−1^ for agricultural land use for soil and food ingestion [56]. In Germany, the maximum permissible level in food is 0.5 mg kg^−1^ [57]. The Government of the United States of America through the National Library of Medicine provides information on Tl toxicity, lethal dose for humans (10–15 mg Tl per kg body weight), and treatment when poison occurs [58]. The European Food Risk Assessment (EU-FORA) prepared a review of food cultivated in areas close to Tl-rich sulfide mineralization, where green cabbage (*B. oleracea* var. *capitata*) is known for its high Tl accumulation; the EU-FORA manifested that the next steps to address Tl toxicity will be to evaluate Tl content in food consumed by the general population and estimate the dietary intake of Tl [59].

Along with other Tl hypertolerant *Brassica* crops, kale clearly has potential for Tl phytoextraction. These crops are fast-growing plants and easily adapt to drought and lack of nutrients [60, 61]. Therefore, they are excellent candidates for phytoextraction due to the economic value of Tl . Phytoextraction is followed by a series of metallurgical processes to extract the target metal(loid)s from the plant biomass [9]. The formation of TlCl_(s)_ crystals in kale could be advantageous, and Tl can be extracted with carboxylic acids [62]. These extraction steps require further investigation, similar to the refining processes already developed for nickel bio-ores, including hydrometallurgy, pyrolysis and/or pyrometallurgy [63].

## Conclusions

This study presents, to our knowledge, for the first time the simultaneous analysis of live plants using multi-modal XFM techniques to unravel the nature of Tl sequestration in kale. Previous research on Tl ionomics in plants using X-ray absorption near edge structure (XANES) revealed the prevalence of Tl(I) in aqueous [64], and solid form [19]. In addition, we found that Tl(I) is associated with Cl forming solid TlCl(_s_) crystals (∼5 µm) in *B. oleracea* var. *acephala* (kale); this information prompts questions about preventing food poisoning due to high Tl concentrations in *Brassica* crops. As Tl is a non-essential element in living organisms, it was postulated that Tl(I) uses K(I) channels to enter plants, as both univalent ions share similar ionic radii [65]. Research showed that higher K concentrations in nutrient solution can inhibit up to 50% Tl uptake in plants [66, 67]. As we found TlCl crystals in kale, we suggest also using different concentrations of Cl (as KCl for example) to assess the effect on Tl uptake or inhibition by plants. Finally, as Tl is used in high-technologies in superconductors and its price has been increasing over time [68], the fast-growing *Brassica* crops are ideal models for exploring metallurgical steps to extract Tl from the harvested plant biomass following phytoextraction.

## Supplementary Material

mfag010_Supplemental_File

## Data Availability

The data underlying this article will be shared on reasonable request to the corresponding author.
