# Supplementary material for: The nature of thallium crystals in Brassica oleracea (kale): a synchrotron multi-technique investigation
Source: Metallomics. 2026 Feb 23;18(1):mfag010. doi: 10.1093/mtomcs/mfag010 (PMC13017095; doi:10.1093/mtomcs/mfag010)
Supplement: mfag010_Supplemental_File [file mfag010_supplemental_file.pdf]

Supplementary Information

The nature of thallium crystals in *Brassica oleracea* (kale): a synchrotron multi-technique investigation

Amelia Corzo-Remigio[1], Hugh H. Harris[2], Michael Jones[3], Tony Wang[3], Dennis Bruckner[4], Kathryn Spiers[4], Jan Garrevoet[4], Antony van der Ent[5*]

[1]Centre for Environmental Responsibility in Mining, Sustainable Minerals Institute, The University of Queensland, Australia.

[2]Department of Chemistry, The University of Adelaide, Australia.

[3]Central Analytical Research Facility, Queensland University of Technology, Australia,

[4]Deutsches Elektronen-Synchrotron DESY, Germany.

[5]Laboratory of Genetics, Wageningen University and Research, The Netherlands.

*Corresponding author: Antony van der Ent (antony.vanderent@wur.nl)

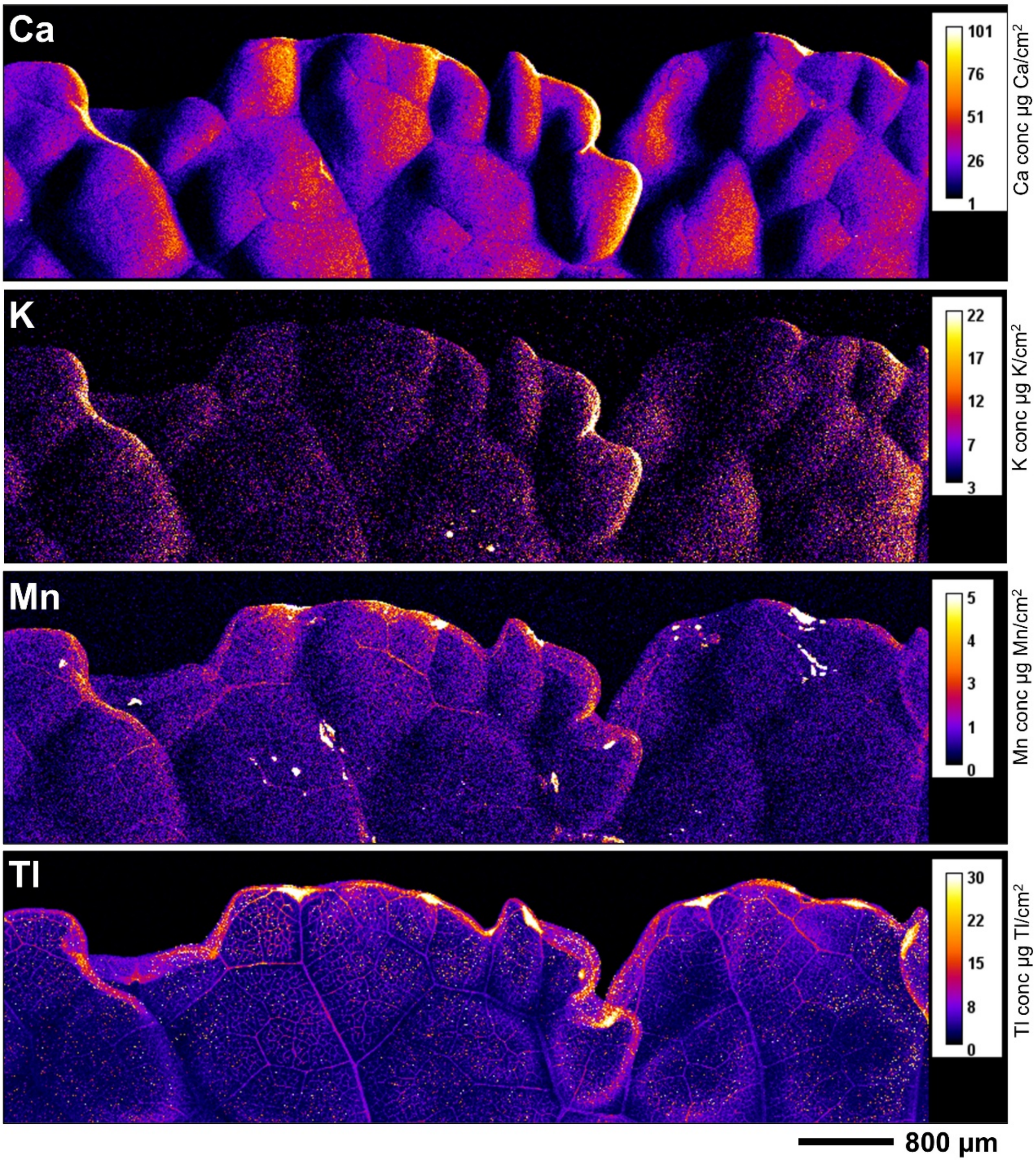

Supplementary Figure 1. Synchrotron-based µXRF elemental maps of Ca, K, Mn and Tl of the border leaf section of *Brassica oleracea* var. *acephala*, kale, Red Russian cultivar. The total acquisition time for the scan was 7 minutes, with a dwell time of 5 ms, and 10 µm resolution. Scale bar denotes 800 µm.

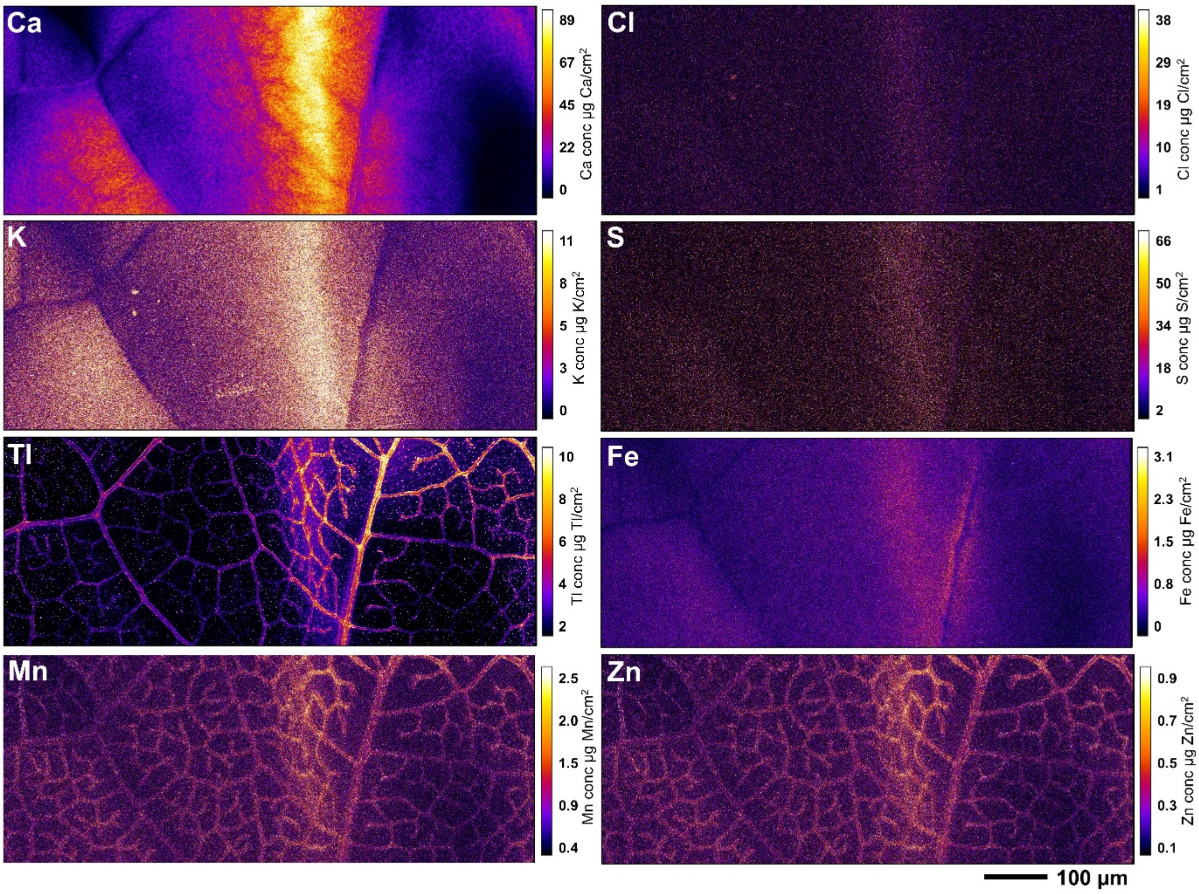

Supplementary Figure 2. Synchrotron-based µXRF elemental maps of Ca, Cl, Fe, K, Mn, S, Tl, and Zn of a leaf section of *Brassica oleracea* var. *acephala*, kale, Red Russian cultivar. The total acquisition time for the scan was 7 minutes, with a dwell time of 5 ms, and 10 µm resolution. Scale bar denotes 100 µm.

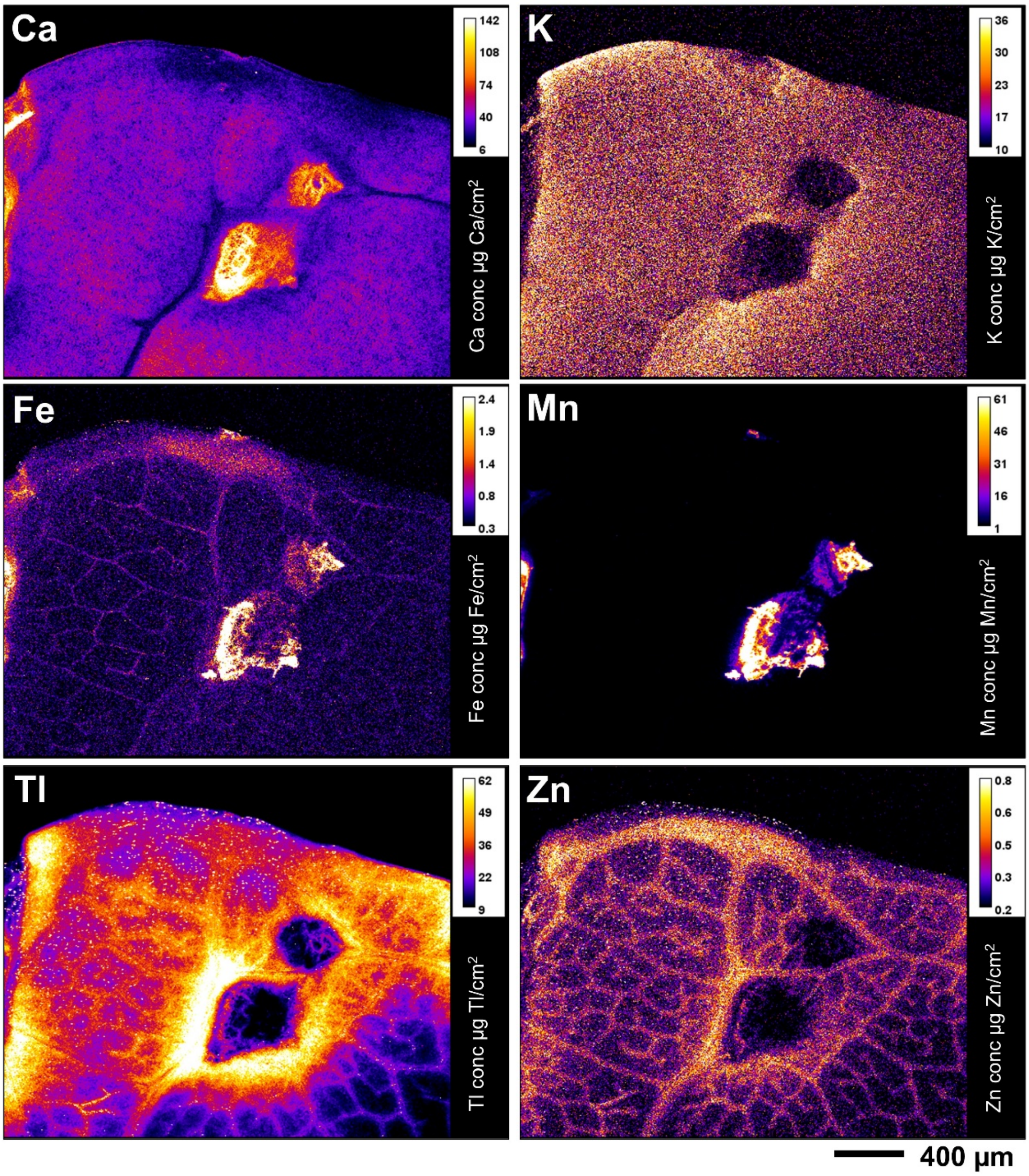

Supplementary Figure 3. Synchrotron-based µXRF elemental maps of Ca, Fe, K, Mn, Tl, and Zn of a leaf section of *Brassica oleracea* var. *acephala*, kale, Red Russian cultivar. The total acquisition time for the scan was 6 minutes, with a dwell time of 5 ms, and 10 µm resolution. Scale bar denotes 400 µm.

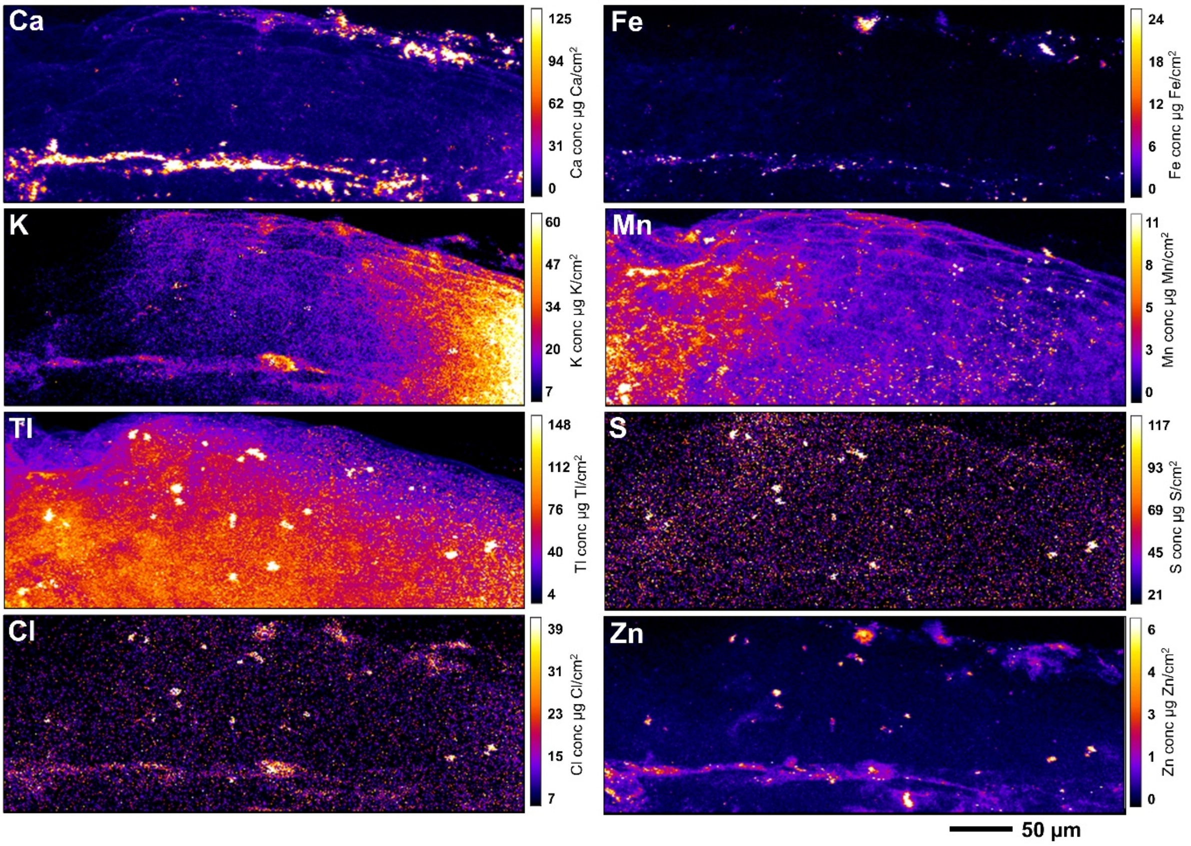

Supplementary Figure 4. Synchrotron-based µXRF elemental maps of Ca, Cl, Fe, K, Mn, S, Tl, and Zn of a leaf section of *Brassica oleracea* var. *acephala*, kale, Nero di Toscana cultivar. The total acquisition time for the scan was 6 minutes, with a dwell time of 5 ms, and 1 µm resolution. Scale bar denotes 50 µm.

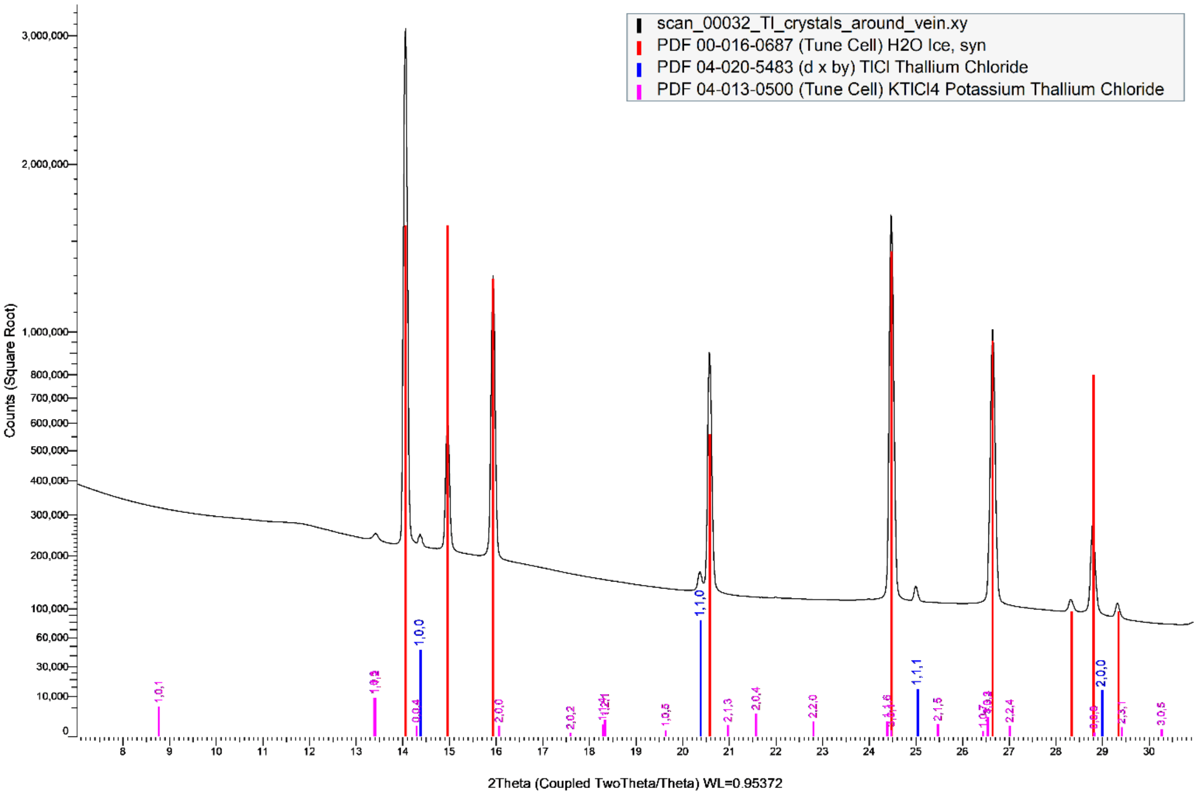

Supplementary Figure S5. Phase Identification of a typical summed XRD pattern.

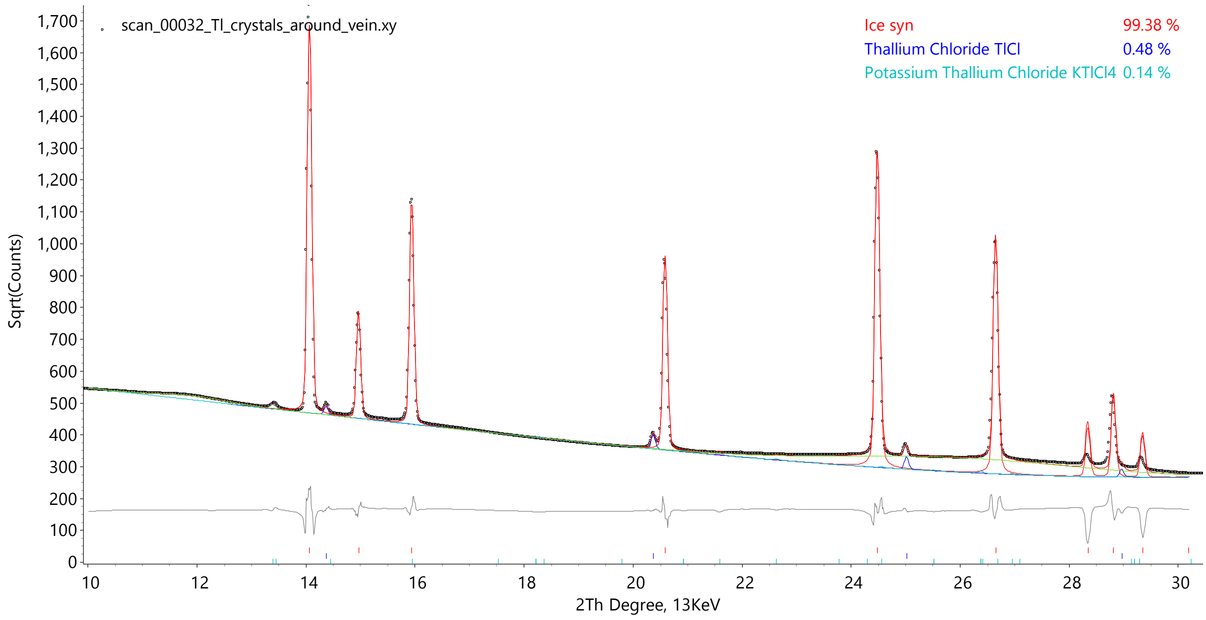

Supplementary Figure S6. Rietveld full pattern refinement conducted using DIFFRAC.TOPAS v7. R_{wp} = 5.73%

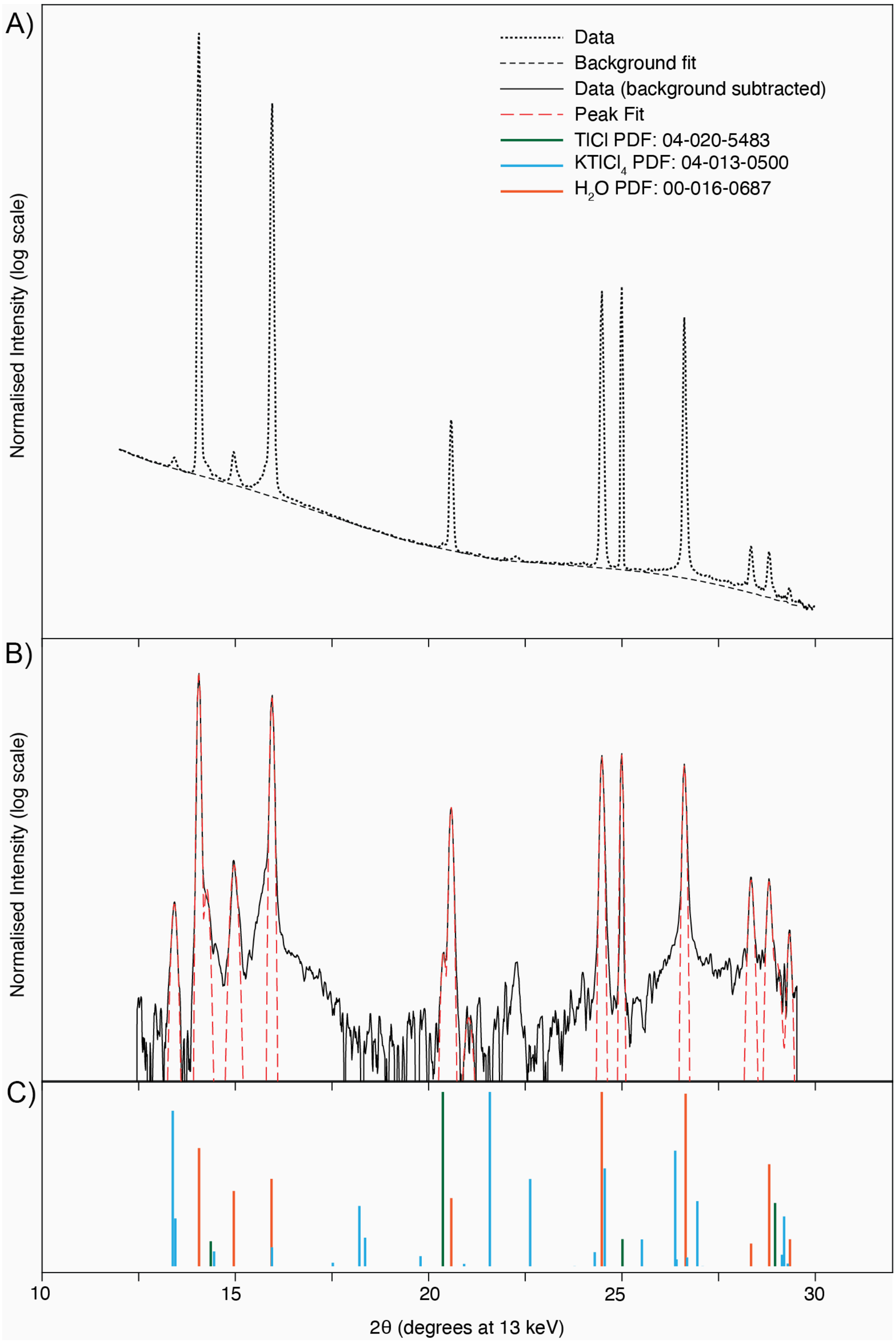

Supplementary Figure S7. Data processing for micro-XRD mapping: A) the background of the azimuthal average of the data for each pixel was removed using the SNIP algorithm with 30 iterations; B) The set of peaks identified in Table 2, along with ice (PDF# 00-016-0687) were fitted with a simultaneous set of Gaussian peaks; C) shows the full set of peaks for the three identified phases.

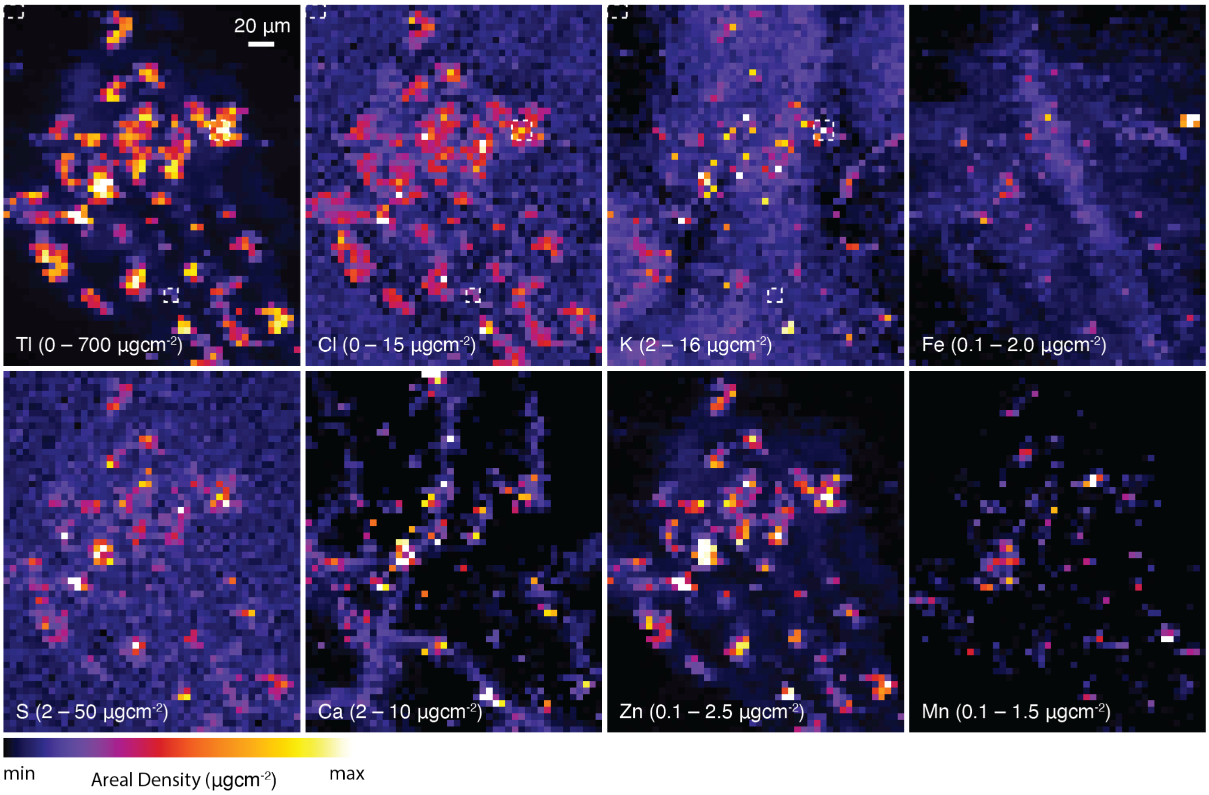

Supplementary Figure S8. Elemental maps corresponding to Figure 4. The total acquisition time for the scan was 3 minutes, with a dwell time of 1000 ms, and 1 µm resolution.

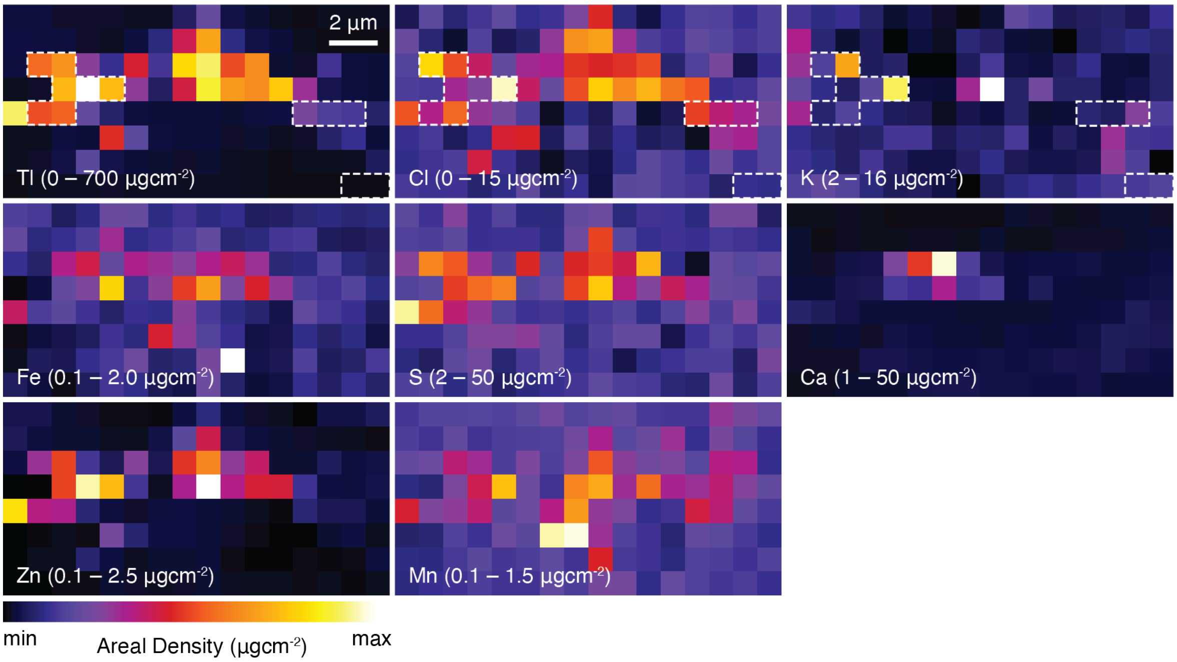

Supplementary Figure S9. Elemental maps corresponding to Figure 5. The total acquisition time for the scan was 55 minutes, with a dwell time of 1000 ms, and 1 µm resolution.

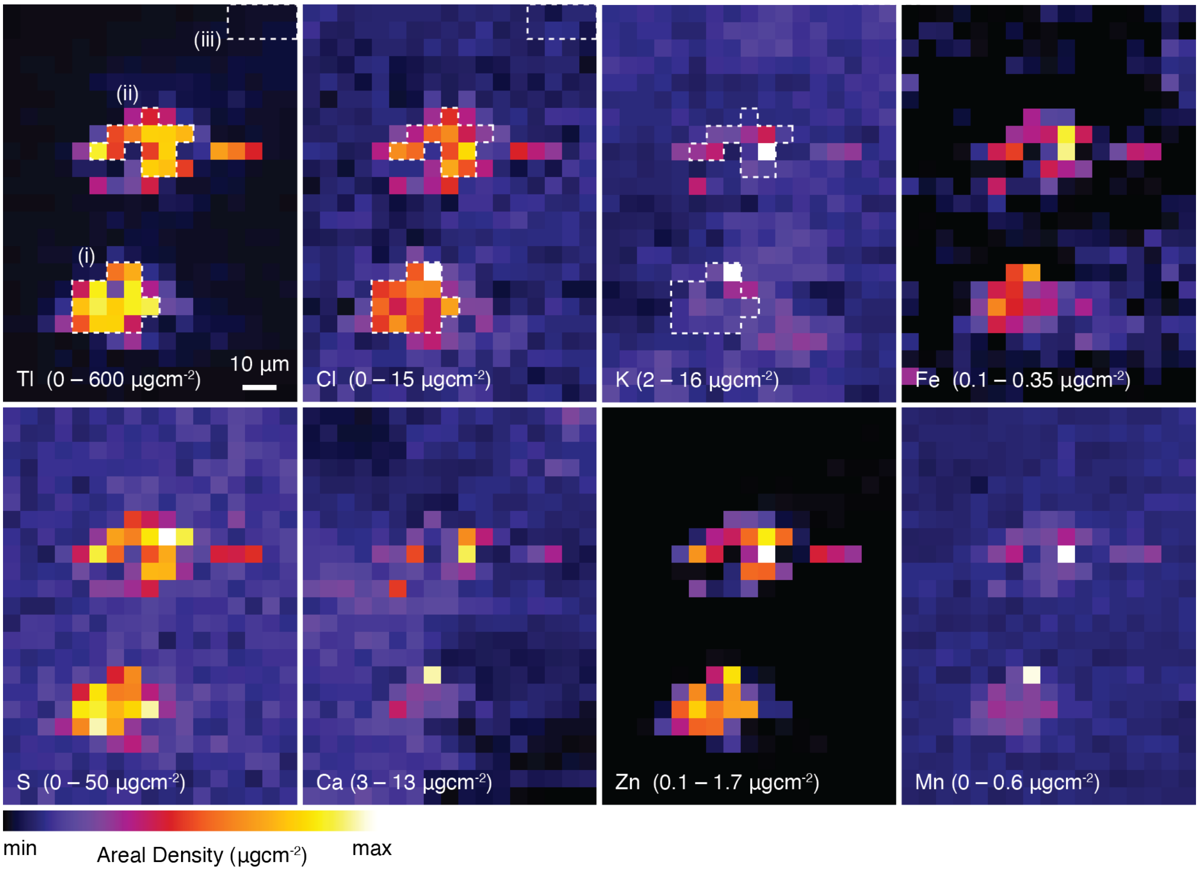

Supplementary Figure S10. Elemental maps corresponding to Figure 6. The total acquisition time for the scan was 7 minutes, with a dwell time of 1000 ms, and 0.5 µm resolution.
